# Identification of modules and functional analysis in CRC subtypes by integrated bioinformatics analysis

**DOI:** 10.1371/journal.pone.0221772

**Published:** 2019-08-30

**Authors:** Ru Chen, Aiko Sugiyama, Hiroshi Seno, Masahiro Sugimoto

**Affiliations:** 1 Department of Gastroenterology and Hepatology, Graduate School of Medicine, Kyoto University, Kyoto, Japan; 2 DSK Project, Medical Innovation Center, Graduate School of Medicine, Kyoto University, Kyoto, Japan; 3 Research and Development Center for Minimally Invasive Therapies Health Promotion and Preemptive Medicine, Tokyo Medical University, Tokyo, Japan; University of Nebraska Medical Center, UNITED STATES

## Abstract

Colorectal cancer is one of the top three causes of cancer-related mortality globally, but no predictive molecular biomarkers are currently available for identifying the disease stage of colorectal cancer patients. Common molecular patterns in the disease, beyond superficial manifestations, can be significant in determining treatment choices. In this study, we used microarray data from colorectal cancer and adjacent normal tissue from the GEO database. These data were categorized into four consensus molecular subtypes based on distinct gene expression signatures. Weighted gene-based protein–protein interaction network analysis was performed for each subtype. NUSAP1, CD44, and COL4A1 modules were found to be statistically significant and present among all the subtypes and displayed though similar but not identical functional enrichment results. Reference of the characteristics of the subtypes to functional modules is necessary since the latter can stay resistant to platform changes and technique noise when compared with other analyses. The CMS4-mesenchymal group, which currently has a poor prognosis, was examined in the study. It is composed mainly of genes involved in immune and stromal expression, with modules focused on ECM dysregulation and chemokine biological processes. Hub genes detection and its’ mapping into the protein–protein interaction network can be indicative of possible targets against specific modules. This approach identified subtypes using enrichment-oriented analysis in functional modules. Proper annotation of functional analysis of modules from different subtypes of CRC might be directive for finding extra options for treatment targets and guiding clinical routines.

## Introduction

Colorectal cancer (CRC) is a complex and heterogeneous disease and has a significant contribution to cancer mortality [1,2,3]. One of the major drawbacks of the treatment of CRC is its heterogeneity, as evidenced by multiple clinical manifestations, mutational profiles, and survival rates. This heterogeneity leads to variability in the efficiency of standard treatment approaches [4]. Traditionally, CRC has been characterized using pathology and clinical phenotype [5,6], which sometimes works well; however, there is usually a delay in the detection prior to the onset of symptoms. There are also problems in distinguishing diseases with complex and overlapping clinical signs.

With the advancement of technology for genomic sequencing, heterogeneity can be identified at a molecular level. Previously, microsatellite instability (MSI) and chromosomal instability were major criteria for molecular classifications. A third molecular subtype was later added to the two well-characterized subtypes, taking into account microsatellite stability and molecularly with a higher level of CpG island methylation. These classifications provided valuable additional information [7]. It became apparent that the newly added subtypes exhibited chemoresistance against epidermal growth factor receptor-targeted therapy [8]. Much more precise classifications of CRC were established using four biologically distinct consensus molecular subtypes: MSI-immune, epithelial and canonical, epithelial and metabolic, and mesenchymal subtypes [9]. These four subtypes cover more than 85% of CRCs.

Despite the promising results of intrinsic subtype analyses, it’s transformation into personalized treatment strategies is still limited. Many patients underwent the same standard care based on their pathological stage or clinical manifestations; however, discovering that these approaches end up with variable survival rates is not uncommon [10]. The application of promising results from research into actual clinical treatment are often delayed [11], leaving many positive discoveries untrialed.

Because response feedback systems for targeted malignancy treatments are rather substandard, the clear division of tumors into subtypes has gained practical importance as a way for researchers to investigate the molecular features of malignancy and identify precise molecular phenotypes [12], which can be used to inform decisions, such as treatment regimens and issues pertaining to medical care. However, the interactions between the genes involved in each subtype of CRC are not fully understood. In particular, enriched functional analysis is a valuable approach. Further, the extent to which processes which are enriched in these networks are related to clinical outcomes is unclear.

PPI network is an original element we adopted in this study, and thesis that it can be informative in the target perspectives was established far early [13], although there is debate on the aspects of whether a drug target protein can be represented as the hub of the PPI network. It was also suggested that multiple specific motifs from the PPI networks can be meaningful in detection of functional dependency of most drugs [14]. Meanwhile, the necessity of mapping drug targets into the integrated biological networks to identify the optimal points of PPI for drug discovery was reported [15]. There are other methodologies applied with PPI network that can be helpful in predicting drug targets and finding hub genes [16], approaching a better profile of interactions among molecular function in the whole system, among which, module screening in the PPI was a must.

Module and function analysis can be important [17]. A module is a stable functional unit in a gene expression set. For example, in breast cancer, therapy choices based on subtypes determined by clinical markers have proven to be effective compared with treatment based simply on the pathological stage [18]. Each subtype can have its own unique functional subnetwork of enriched genes. However, it is not known how the recently determined consensus CRC molecular subtypes relate to clinically relevant pathological subtypes and treatment choices [19]. The objective of this study is to discover enriched functional modules in each subtype of CRC, with the aim of better identification of the variances at the molecular level. A crucial element of the study was the use of unsupervised modules, which are robust to noise and tend to identify at least a few member genes represented across multiple platforms. To the best of our knowledge, this is the first attempt to investigate modules for separate subtypes of CRC since the establishment of consensus molecular subtype of CRC.

In this study, we analyzed biological functional modules and provided evidence that targeted treatment selection based on modules at the molecular level can be realized as subtypes of CRC and may be valuable for developing integrated models that can predict clinical outcomes.

## Materials and methods

### Study design

**Fig 1:**
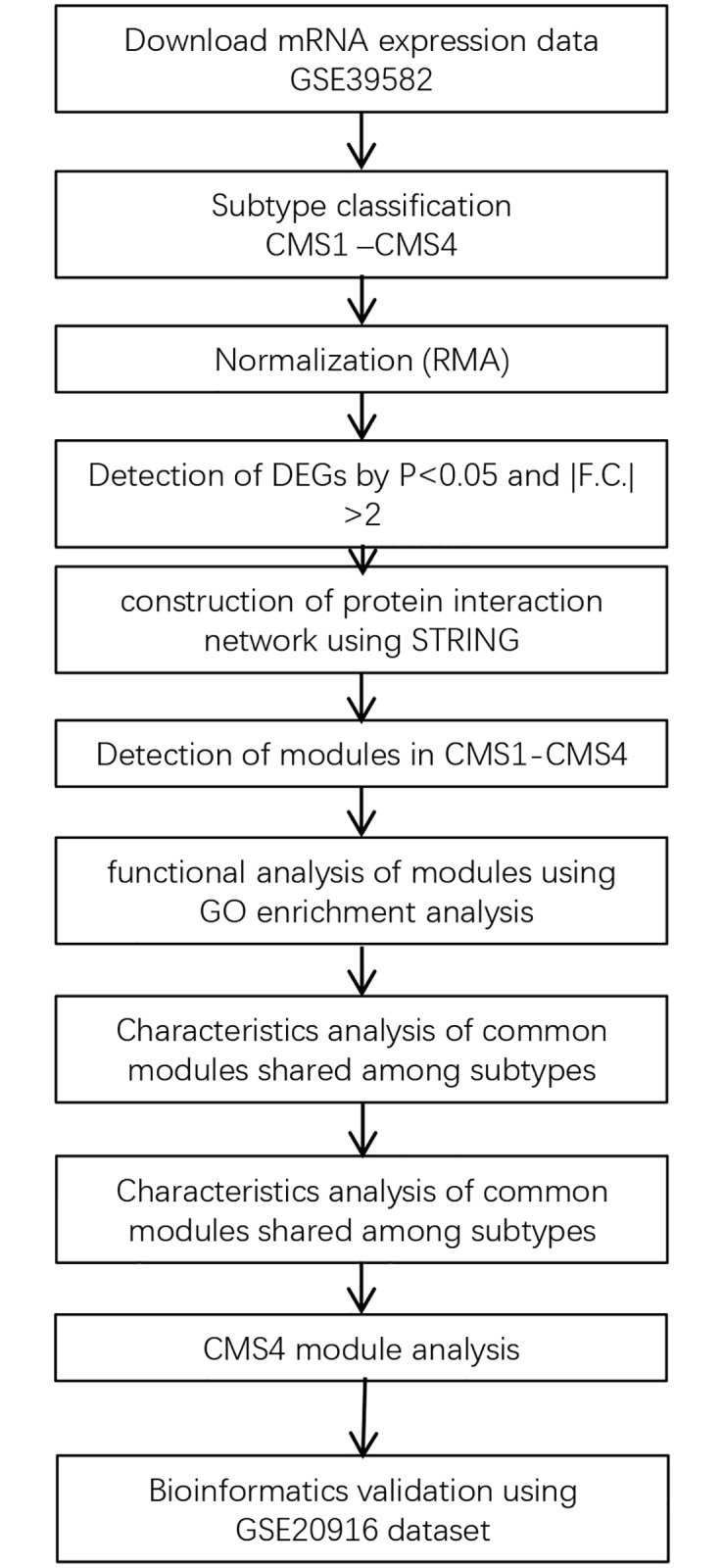
Schematic workflow of the bioinformatics pipeline used for the search of significant modules in this study.

This study focused on discovering functional modules in a CRC molecular network. The raw data from CRC samples was classified into four categories based on consensus molecular subtypes, then compared with normal samples. We used gene expression data from CRC samples. Differentially expressed genes (DEGs) were categorized into upregulated and downregulated groups, and network inference algorithms were used to construct protein–protein interaction (PPI) networks, which were visualized using Cytoscape [20]. Significant modules in each part of the CRC subnetwork were studied, with the aim of identifying promising targetable points inside CRC modules. A complete workflow for this study is depicted in Fig 1.

### Microarray data

The CRC gene expression profiles used in this study were downloaded from the NCBI database with the accession number GSE39582, which was processed using the Affymetrix HGU133A platform, and contained 462 samples, including 443 CRC samples and 19 normal colon samples. Statistical analysis of the GEO dataset was performed using R (Version 1.1.453). All raw data were normalized and converted to log_2_ ratio format using the robust multichip average algorithm [21]. RMA algorithm was often applied in generating matrix from gene chip and microarray data, which consisted of three sections in dealing with large amount of data: background subtraction, quantile normalization and summarization, and exceeded in preparing data for numerous downward R packages analysis.

### Identification of subtypes

The analysis of the raw CRC data was carried out using the R package CMScaller [22], which implements an algorithm optimized for the comparison of consensus molecular subtypes. CRC data were divided into four consensus molecular subtypes, and gene expression data that were excluded in the four subtypes were removed from this study.

### DEGs

The DEGseq R package [23], in which SAM algorithm is kernelled, was used to detect DEGs with an absolute log fold change (FC) > 2, and a *P*-value < 0.05 was considered to be statistically significant. DEGs were used to sort genes with upregulated and downregulated expression in each CRC subtype data vs. the normal colon epitheliums.

### Integration of PPI network and module detection

The PPI network was created using the online database STRING (Search Tool for the Retrieval of Interacting Genes) [24], which holds data about known and predicted protein–protein interactions. DEGs in each subtype were mapped using this tool. The criterion for retaining interactions was a combined score > 0.4. The Cytoscape plug-in Molecular Complex Detection (MCODE) was used to identify modules in the PPI networks. The criteria for the identification of significant modules were MCODE score > 3 and number of included nodes > 4.

### Functional analysis of modules

All modules identified for each consensus molecular subtype were examined for overrepresented GO categories [25,26,27]. The analysis of Gene Ontology term enrichment (GO) is widely used for interpreting the biological significance of sets of genes and the processes in which they are involved. The DAVID database [28] was used to map genes from modules to detect the relevant biological annotations of GO terms. A *P*-value < 0.05 was considered to be statistically significant. The PPI networks of CMS4 can also be visualized using REVIGO [29], which helps to unveil the inner connections of all CMS4-enriched biological processes.

### Survival analysis of hub genes

The HCAR3 module of CMS4 was selected for further analysis. This module included 32 genes. The top ten ranked genes determined by the number of interactions it formed in this module were selected as the signature gene set representative of the module. The relationship between recurrence-free survival and possession of the HCAR3 module signature gene set was assessed using Cox regression survival analysis in SurvExpress [30], and Kaplan–Meier survival plots stratified by these ten genes were constructed.

### Validation of the workflow using bioinformatic approach

The applicability of this workflow in our study was partially examined using a bioinformatic approach with another dataset from the GEO database, with the accession number of GSE20916. This dataset included both malignant samples and paired normal tissue. DEGs were screened by comparison with normal tissue gene expression levels, following the identification of CMSs. Modules with a high proportion of enriched processes were investigated in depth in CMS4.

## Results

### Identification of DEGs

**Fig 2:**
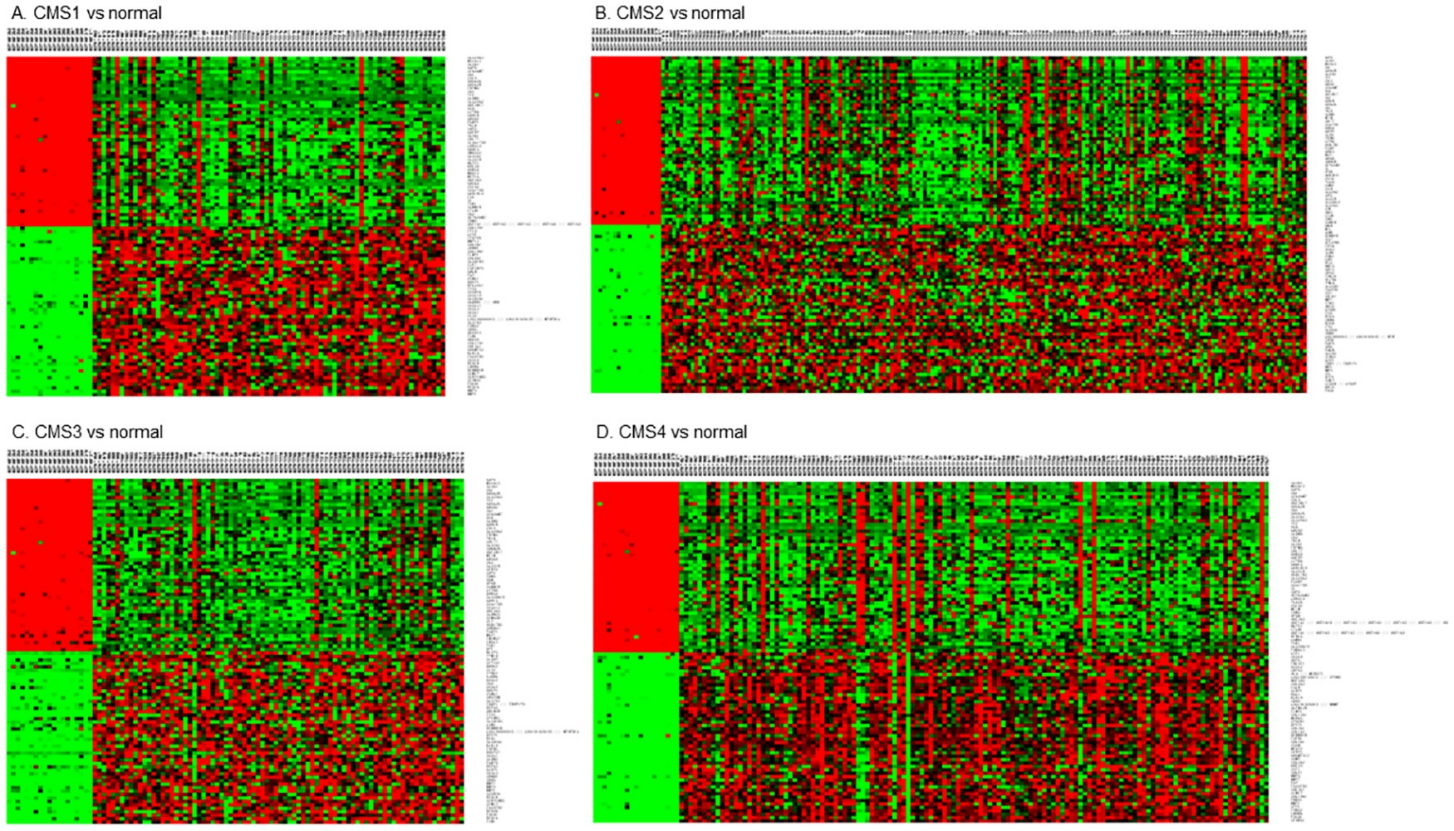
Heat maps visualization of the pattern of expression change for the top 50 differentially expressed genes. The upper row of each heat map consisted of the detailed configuration of the left 19 normal samples and corresponding CRC subtype samples. The right column was the 50 up-regulated and 50 down-regulated DEGs among each subtype *vs* normal samples, represented in red and blue respectively in the map. Software meV. (version 4.7, http://mev.tm4.org/) was used for heat map display.

Using a CMS classification of GSE39582, a total of 2930, 2846, 2286, and 2627 DEGs were identified in CMS1, CMS2, CMS3, and CMS4 for each subtype, respectively. Heat maps of the 50 top-ranked genes with respect to DEGs expression in each subtype are shown in Fig 2(A)–2(D).

### PPI network construction and module analysis

**Fig 3:**
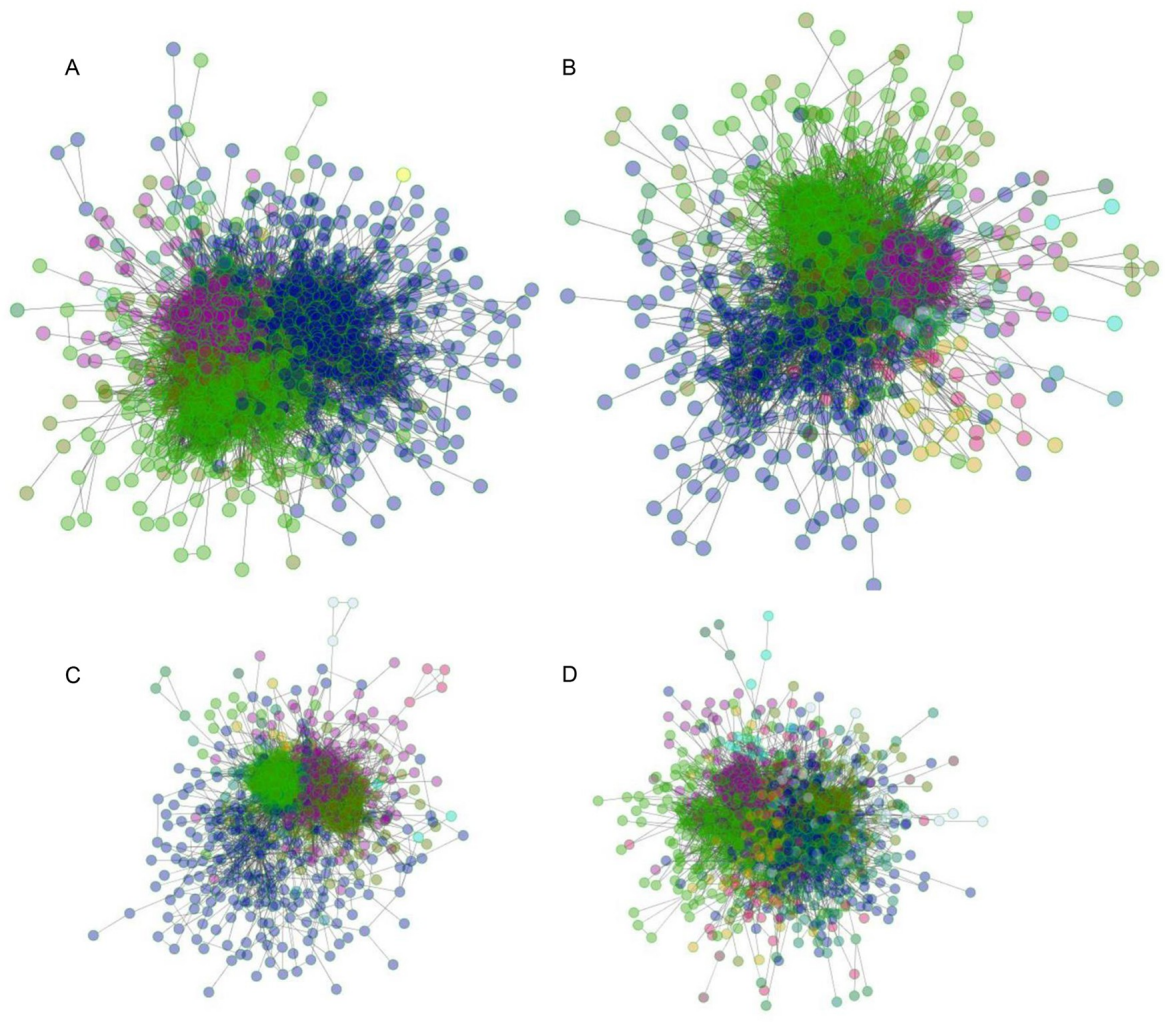
Construction of PPI and module networks in CRC subtypes. Nodes of a module forming subnetworks of PPI were colored differently on a subtype basis (same color across different subtypes didn’t represent similar groupings of module function). The images of CMS1(A), CMS2(B), CMS3(C), CMS4(D) were shown accordingly.

DEGs in each subtype were mapped using the STRING database to construct a PPI network with a total of 2163 nodes and 23939 edges in CMS1, 2163 nodes and 23939 edges in CMS2, 1699 nodes and 16578 edges in CMS3, and 1974 nodes and 12939 edges in CMS4. The modules identified for each subtype are shown in Table 1, which briefly summarizes some of the key differences in modules between subtypes. The number of significant modules for each subtype varied, and the structure of the networks for each subtype differed in node composition and number of links in a component. The number of links in a module is the number of connections between nodes and represents the interactivity of the component. In Fig 3, we show a visualization of the PPI network for each subtype, with nodes colored based on modules. It is easy to observe the contrasts in the architectures of the PPI network between each network subtype. Both the number of colors and the structure of each module were unique for each CRC subtype.

**Table 1:** Module configuration in each subtype.

DEGs	Subtypes	Number of Connected Components of Significant Modules	Number of Modules of Significance	Number of Nodes in the Largest Component	Number of Links in the Largest Component
**Up-Regulated**	CMS1	7	19	122	6347
	CMS2	5	20	109	5145
	CMS3	5	15	106	4925
	CMS4	9	16	48	1074
**Down-Regulated**	CMS1	16	17	72	601
	CMS2	11	20	21	210
	CMS3	12	18	21	210
	CMS4	12	14	36	301

### GO terms are associated with modules for each CRC CMS

All of the components of each subtype were analyzed using GO term enrichment (Table 2). Each module was named after the top-ranked gene. Consistent with the results shown in Fig 3, most of the enriched modules varied across subtypes, with respect to the number of enriched modules and the gene composition in the modules, as well as the number of processes observed to be enriched in each module. Gene composition was not directly correlated with the number of enriched processes in a module. For instance, the IFITM2 module from CMS3 contained only four genes, yet ten processes were enriched in this module; in contrast, the RSL1D1 module from the same subtype, which was composed of 38 genes, had only three enriched processes.

**Table 2:** Enriched modules (score>3) found in each subtype network.

Subtypes	Number of Enriched Modules	Module that Enriched	Number of Genes	Number of Enriched Processes
**CMS1**	15	NUSAP1	122	49
		COL4A1	22	11
		XPO1	36	19
		HCAR3	56	25
		CEP152	38	19
		SNRPB	35	55
		IFIT3	9	3
		SPP1	26	9
		IL8	42	3
		CASC5	19	1
		CD44	21	6
		CSGALNACT2	5	6
		BMP7	10	25
		SCD	6	2
		NMB	14	4
**CMS2**	15	NUSAP1	109	37
		RSL1D1	78	16
		COL4A1	18	3
		POM121C	15	24
		CCT4	42	30
		PPIL1/PRPF4	27	7
		MTHFD1	22	1
		BRCA1	22	8
		EXOSC8	11	16
		MRE11A/SKP2	8	1
		WNT5A/AXIN2/WNT2	10	3
		CD44	10	10
		SCD	5	4
		PLCB1	11	1
		MRPL36	24	4
**CMS3**	12	NUSAP1	106	31
		RSL1D1	38	2
		CXCL2/CXCL1	13	11
		IMPDH2	23	3
		BMP7/COL4A1/PLCB1	20	6
		SNRPB	14	23
		TIMELESS/RFC3/BRCA1	21	6
		CPS1/MTHFD1	13	1
		BRCA2	8	1
		SHH/CD44	15	18
		IFITM2	4	10
		PAICS	10	2
**CMS4**	12	NUSAP1	48	29
		COL4A1	27	8
		HCAR3	32	32
		CD44/SPP1	20	9
		ENG	35	6
		HSPG2	31	2
		CSGALNACT2	6	5
		SNRPB	5	2
		GPC6	19	10
		GART	36	11
		IFITM2/FLNA	19	9
		ENTPD1	4	1

### NUSAP1, CD44, and COL4A1 are present in all four consensus molecular subtypes

**Fig 4:**
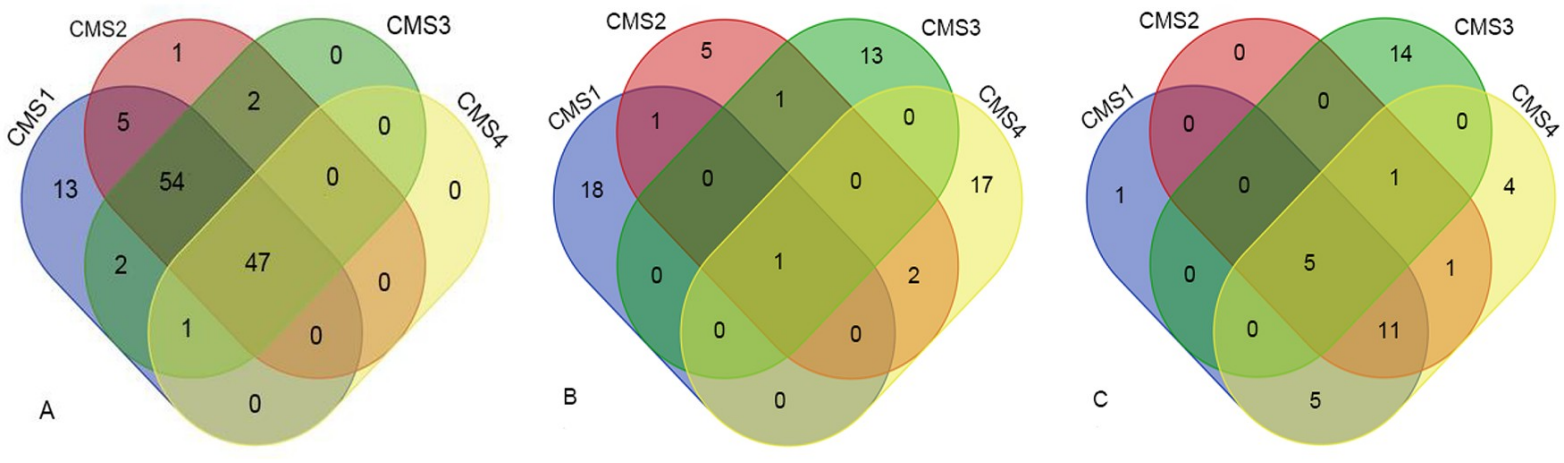
Venn diagram of overlapping results of gene compositions among subtypes. Overlaps of gene organization of three modules from each molecular subtype were shown as NUSAP1(A), CD44(B) and COL4A1(C); a number represented the number of genes shared between/among subtypes or unique to its’ own.

NUSAP1, CD44, and COL4A1 are present in all subtypes. NUSAP1, the gene coding for nuclear and spindle-associated protein 1, plays an important part in the process of spindle microtubule organization; CD44 encodes a cell-surface protein that is involved in cell–cell interaction; COL4A1 is the gene for collagen alpha-1(IV), a flexible protein that provides instructions for making integral components of the basement membrane. The gene composition of the three modules shared few similarities besides overexpression pattern. The Venn diagram shown in Fig 4 depicts the gene composition of NUSAP1(A), CD44(B), and COL4A1(C) for each CRC subtype.

The NUSAP1 module had the same 47 genes among subtypes, with 21 enriched processes shared across subtypes, most of which included protein phosphorylation, ubiquitin-protein ligase activity, and microtubule-based movement processes. In the CD44 module, gene composition shared little resemblance between subtypes and was dependent on each subtype. Two common processes are enriched in the COL4A1 module among all subtypes: ECM structural constituent and metal ion binding.

One of the significantly enriched processes in the CD44 module of CMS3 was negative regulation of canonical Wnt signaling pathway. CMS3 is often referred to as the subtype with the least variance; thus, the Wnt pathway tended to be the canonical transduction signal pathway overstimulated in the progression of CMS3.

### Analysis of modules in CMS4

**Fig 5:**
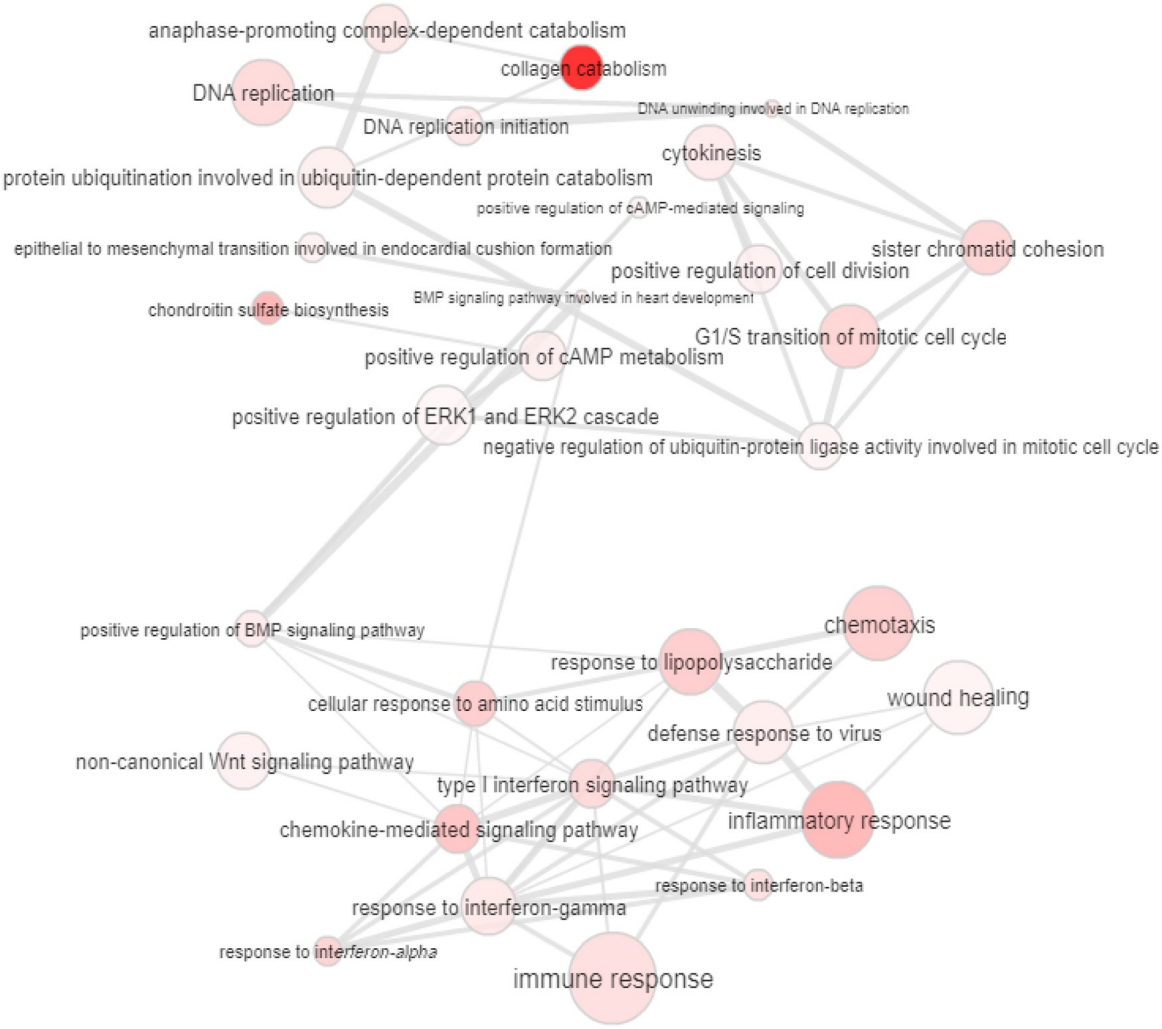
Interactive relationships among enriched GO terms processes in CMS4. The color and size of the nodes indicated the input p value and the frequency of the GO term generated from GOA database; edges that linked nodes in the graph indicated similarity.

There were 16 modules detected in CMS4, of which 12 were enriched. All of the enriched biological processes were evaluated using REVIGO, as shown in Fig 5. It is clear that the modules with the most interacting enriched processes consist of inflammatory and immune response and apoptosis processes. This may indicate that although numerous sets of nodes model modules, there can be stronger interactions formed between certain modules, as in the case of the HCAR3 module, which was the only module in which the chemokine-associated process was observed.

### HCAR3 module and mesenchymal invariance

**Fig 6:**
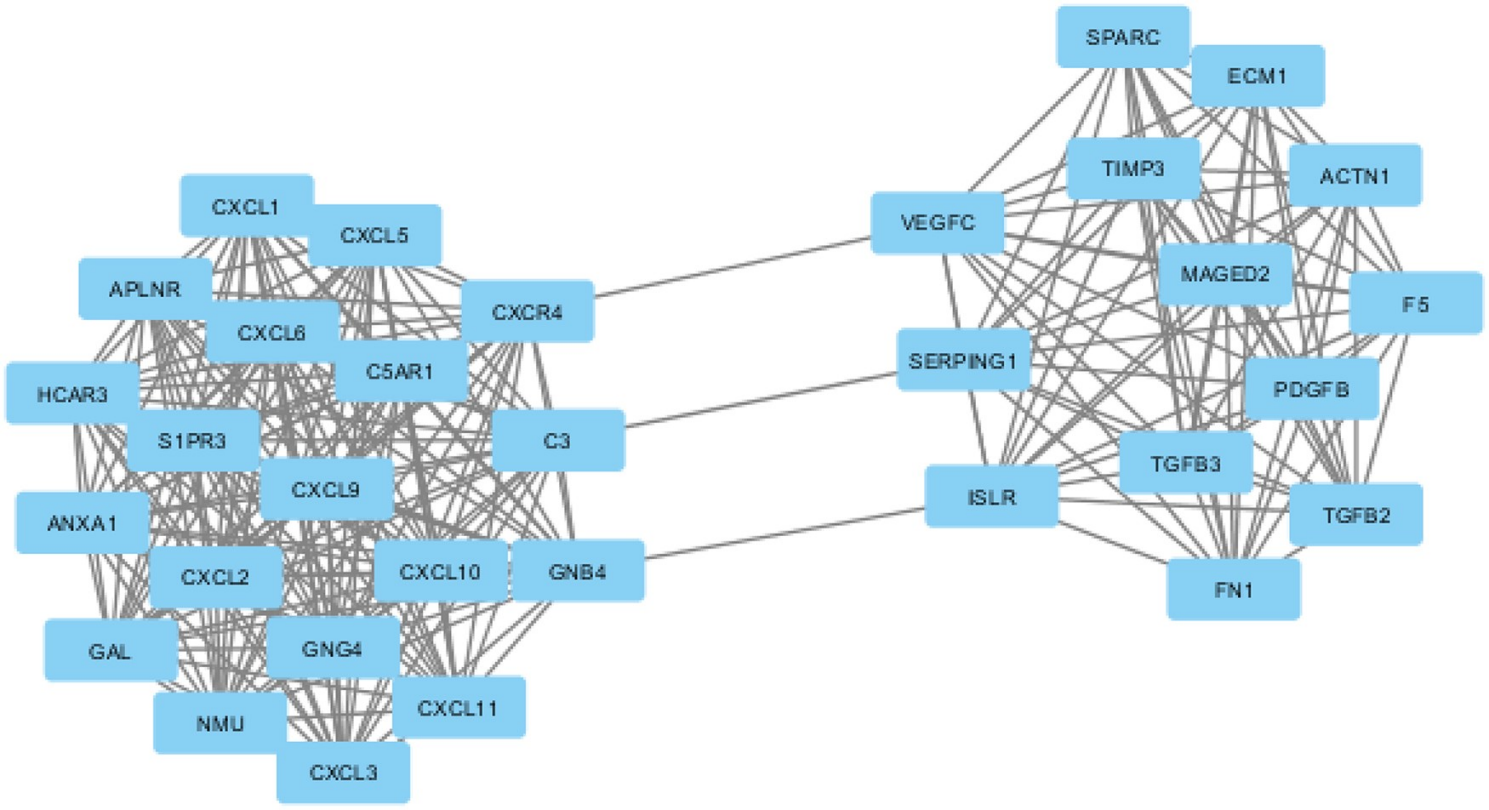
HCAR3 module contained two subgroups. The color and size of the nodes indicated the input p value and the frequency of the GO term generated from GOA database; edges that linked nodes in the graph indicated similarity.

As seen in Fig 6, there were two subgroups in the HCAR3 module of CMS4, centered on ECM dysregulation and chemokine-associated processes. Subgroup centralities in the HCAR3 module indicated that some genes related to functional variance and coordination overview were involved. The tightly concentrated module of chemokine-associated processes and the ECM dysregulation process are often linked together as common mesenchymal traits in malignancy, with a synchronized mutual regulation.

### The HCAR3 module is associated with survival in CRC

**Fig 7:**
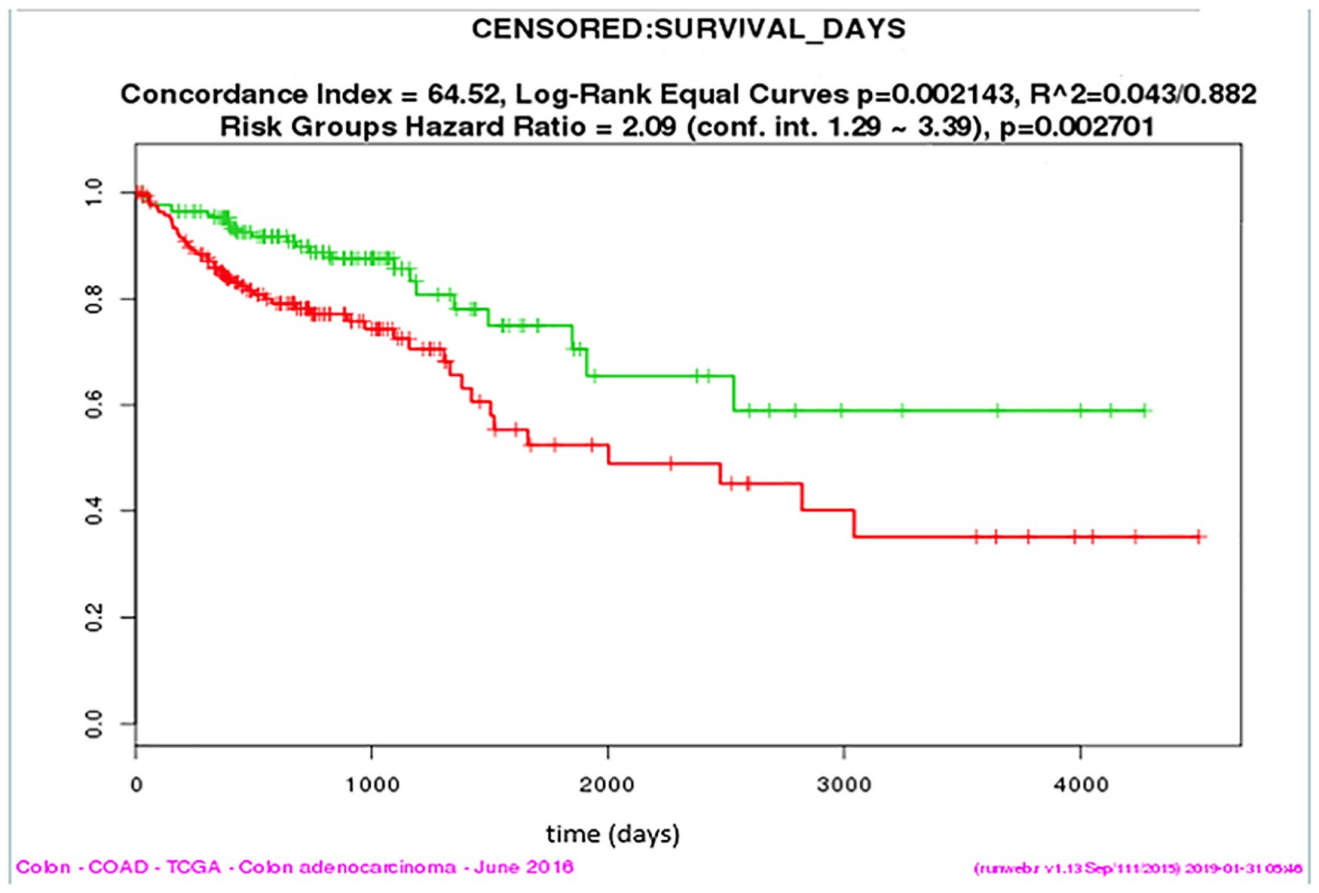
Kaplan–Meier Plotter of relapse-free survival evaluating genes expressed in HCARS module from CMS4 using TCGA COAD clinical data. Red curve represented high expression level and indicated the poor prognosis, while green curve represented low expression level of the prognosis signature.

Using TCGA COAD clinical data as references, survival analysis of every gene contained in the HCAR3 module was performed. No significant differences were observed using single-gene analysis; however, survival analysis using multiple genes from the HCAR3 module indicated that colorectal cancer patients with higher expression of multiple genes from HCAR3 module had worse survival outcomes (Fig 7). As a potential new signature in the context of CRC survival analysis, the hazard ratio was 2.09, indicating a worse hazard of death from the patients possessing these genes. A Kaplan–Meier curve was generated, the log rank *P*-value of which was 0.002143 and the concordance index 0.64, suggesting a relatively good interpretation of survival prediction.

### Bioinformatic validation of possible biomarkers

DEGs generated from comparisons of cancer samples in each subtype and 24 paired normal tissues were further mapped to the PPI network; the details are shown in S1 Table. In CMS4, the IL-6 module had a relatively higher number of enriched processes compared with the number of genes included in the module. Thirteen of the 22 genes in this module were part of the HCAR3 module of CMS4 in the analysis of GSE39582. Among them, genes with high weight included several from the CXCL family and the HCAR3 gene. Survival analysis of multiple genes in the IL-6 module was performed (S1 Fig) confirming the possible value of modules as biomarkers.

## Discussion

Efforts have long been made to understand the heterogeneity of CRC, starting with the initial polymerase chain reaction-based method, in order to establish effective treatment strategies. Previous subtypes have, to some extent, achieved this purpose [31]. For example, MSI+ tumors, which have genomic mutation and exhibit immune cell infiltration, respond well to chemotherapy, including immune checkpoint inhibitors. CMS classification is the method applied in this study and is formed through the merging of information from weighted analyses of major subtypes as classified at that time. Retrospective analysis of clinical trials, for instance, those involvingCMS4 with a mesenchymal-like phenotype which benefits less from chemo-regimes containing anti-EGFR ingredients, has demonstrated the potential predictive value of the CMS classification.

Three genes, NUSAP1, CD44, and COL4A1, were shared among the four subtypes identified, despite their molecular differences. NUSAP1 plays an important role in regulating BRCA1 protein levels [32] and is reported to be a predictor of poor prognosis in CRC [33]. In our findings, the expression level of the NUSAP1 module was upregulated in all CRC subtypes. Even though gene composition varied among different subtypes, they were involved in similar processes, such as mitosis and microtubule movement dysfunction. CD44, which is a well-known immune membrane factor, coordinating alteration of signal expression during the progression of malignancy, acts as a “marker molecule” in the process of EMT: a common process for majority of malignant transformations [34]. COL4A1, a tumor angiogenesis indicator, is regulated by the p53 gene and functions in association with endothelial cells with destabilized matrix [35]. All three genes have vital roles to play in CRC progression.

CMS4 is the only subtype in which adjacent mesenchymal expression is involved. Often, patients diagnosed with CMS4 have the worst prognosis. In this study, CMS4 had 16 modules after PPI network screening, of which 12 were statistically significant in Gene Ontology enrichment analysis. Processes concerning inflammation, the immune system, and apoptosis are the most highly enriched categories. Among all the enriched modules, the HCAR3 module consisted of 32 genes but had relatively higher number of enriched processes per gene compared with other modules, although the HCAR3 gene alone was not a significant factor in the survival of CRC patients. The ten top-ranked genes in this module were shown to be essential in the prediction of survival. Patients with upregulated expression of these genes have lower rates of survival. Most of the ten genes share a gene signature common to metastatic traits: ECM1 is expressed throughout the intestine, and overexpression of ECM1 suggests malignant epithelial cancer as in CRC [36]; CXCL2 [37] and CXCL1 [38] mediate metastatic processes; cells with CXCR4 upregulation shows less sensitivity toward radiotherapy [39]; ACTN1 [40] and S1PR3 [41] aid in invasion enhancement [36]; and C5AR1 can increase cell permeability [42]. In contrast, CXCL9 has been shown to predict good outcomes for cancer patients [43]; HCAR3 and GNG4 exhibit suppressor effects on the process of tumorigenesis in mammary cancer [44, 45] and in GBM [46]. The function of HCAR3 in CRC remains to be elucidated; however, a member of the same HCA receptor family with similar structure, named, HCAR2, which functions as a tumor suppressor gene, has been reported to have reduced expression levels in colorectal cancer cell lines [47]. In this study, HCAR3 was one of the most upregulated genes, forming the largest amount of interactions inside a module, so it is worth investigating as a potential novel target for a drug acting on oncogenic or suppressor processes.

Many bioinformatic pipelines exist, based on different assumptions and algorithms, and produce varying results. Results generated via the methodology applied in this study can be combined with those of other bioinformatic workflows and experimental conclusions to join information from a range of origins to build a solid framework for exploring these issues. The modules in the CMSs of CRC have not been explored in detail, but attempts have been made in studies using weighted correlation network analysis (WGCNA) on CRC microarray data [48], and recurrence-associated modules have been identified.

In the validation section, in which a different expression dataset was used, similar results were found during the detection of CMS4 modules. The major gene composition had a similar trend as the HCAR3 module at the level of enriched functional processes. The CXCL family has long been reported as prognostic biomarkers in colon cancer [49], being involved with inflammatory processes, including genes such as IL-6, C5AR1, and C3 in the IL-6 module. We applied strict rules on the use of datasets in this study; they must have cancer samples that are paired with normal tissue. In this way, we minimized the effects of timing and different data sources.

There is an urgent need for the elucidation of key oncogenic modules to provide an unbiased molecular classification of CRC in order to help tailor treatment choices in the future. In our research, not only modules but also the composition of modules varied among CRC subtypes. Despite these variances, NUSAP1, CD44, and COL4A1 were present in all subtypes. They share processes related to protein phosphorylation, ubiquitin-protein ligase activity, microtubule-based movement, ECM structural constituent, and metal ion binding, all of which are key pathways involved in the progression of malignancy. In CMS4, ten genes from the HCAR3 module are associated with the prediction of patient survival, and the HCAR3 gene is especially important, as it is engaged in multiple interactions and thus might be worthy of further attention with respect to the development of drugs against colorectal cancer.

## Supporting information

S1 Table (XLSX)

S1 Fig (TIF)

## References

[1] Ferlay J, Soerjomataram I, Dikshit R, Eser S, Mathers C, Rebelo M et al Cancer incidence and mortality worldwide: Sources, methods and major patterns in GLOBOCAN 2012. Int J Cancer 2015;136: E359–E386. 10.1002/ijc.29210 25220842

[2] Blanco-Calvo M, Concha Á, Figueroa A, Garrido F, Valladares-Ayerbes M. Colorectal cancer classification and cell heterogeneity: A systems oncology approach. Int J Mol Sci. 2015;16: 13610–13632. 10.3390/ijms160613610 26084042 PMC4490512

[3] Budinska E, Popovici V, Tejpar S, D'ario G, Lapique N, Sikora KO, et al Gene expression patterns unveil a new level of molecular heterogeneity in colorectal cancer. J Pathol 2013;231: 63–76. 10.1002/path.4212 23836465 PMC3840702

[4] Punt CJ. From tumour heterogeneity to advances in precision treatment of colorectal cancer. Nat Rev Clin Oncol. 2017;14: 235–246. 10.1038/nrclinonc.2016.171 27922044

[5] Gonzalez-Pons M, Cruz-Correa M. Colorectal cancer biomarkers: Where are we now? Biomed Res Int. 2015; 27. 10.1155/2015/149014 PMC4461726 26106599

[6] Worthley DL, Leggett BA. Colorectal cancer: Molecular features and clinical opportunities. Clin Biochem Rev. 2010;31: 31–38. 20498827 PMC2874430

[7] Kim JH, Kang GH. Molecular and prognostic heterogeneity of microsatellite-unstable colorectal cancer. World J Gastroenterol. 2014;20: 4230–4243. 10.3748/wjg.v20.i15.4230 24764661 PMC3989959

[8] Inamura K. Colorectal cancers: an update on their molecular pathology. Cancers 2018;10: 26. 10.3390/cancers10010026 PMC5789376 29361689

[9] Guinney J, Dienstmann R, Wang X, De Reyniès A, Schlicker A, Soneson C, et al The consensus molecular subtypes of colorectal cancer. Nat Med. 2015;21: 1350–1356. 10.1038/nm.3967 26457759 PMC4636487

[10] Han Y, Lu S, Yu F, Liu X, Sun H, Wang J, et al A comparative analysis and guidance for individualized chemotherapy of stage II and III colorectal cancer patients based on pathological markers. Scientific Reports. 2016;6: 37240 10.1038/srep37240 27845412 PMC5109035

[11] Han Y, Lu S, Yu F, Liu X, Sun H, Wang J, et al High hospital research participation and improved colorectal cancer survival outcomes: a population-based study. Gut. 2017; 66: 89–96. 10.1136/gutjnl-2015-311308 27797935 PMC5256392

[12] Dawson H, Lugli A, et al Molecular and pathogenetic aspects of tumor budding in colorectal cancer. Front Med., 2015;2: 11. 10.3389/fmed.2015.00011 PMC4354406 25806371

[13] Feng Yanghe, Wang Qi, and Wang Tengjiao. Drug Target Protein-Protein Interaction Networks: A Systematic Perspective. Biomed Res Int. 2017; 6 11. 10.1155/2017/1289259 PMC5485489 28691014

[14] Overington J. P., Al-Lazikani B., Hopkins A. L. How many drug targets are there? Nat Rev Drug Discov. 2006;5(12):993–996. 10.1038/nrd2199 17139284

[15] Hopkins A. L. Network pharmacology: the next paradigm in drug discovery. Nat Chem Biol. 2008;4(11):682–690. 10.1038/nchembio.118 18936753

[16] Zhao Shiwen, Li Shao. Network-Based Relating Pharmacological and Genomic Spaces for Drug Target Identification. Plos One. 2010; 7 26. 10.1371/journal.pone.0011764 PMC2909904 20668676

[17] Qu X, Xie R, Chen L, Feng C, Zhou Y, Li W, et al Identifying colon cancer risk modules with better classification performance based on human signaling network. Genomics 2014;104: 242–248. 10.1016/j.ygeno.2013.11.002 24239682

[18] Godone RL, Leitão GM, Araújo NB, Castelletti CH, Lima-Filho JL, Martins DB. Clinical and molecular aspects of breast cancer: Targets and therapies. Biomedicine & Pharmacotherapy 2018;106: 14–34. 29945114 10.1016/j.biopha.2018.06.066

[19] Schlicker A, Beran G, Chresta CM, McWalter G, Pritchard A, Weston S, et al Subtypes of primary colorectal tumors correlate with response to targeted treatment in colorectal cell lines. BMC Med Genomics 2012;5: 66 10.1186/1755-8794-5-66 23272949 PMC3543849

[20] Shannon P, Markiel A, Ozier O, Baliga NS, Wang JT, Ramage D, et al Cytoscape: a software environment for integrated models of biomolecular interaction networks. Genome Research 2003 13(11):2498–504. 10.1101/gr.1239303 14597658 PMC403769

[21] Irizarry RA, Hobbs B, Collin F, Beazer-Barclay YD, Antonellis KJ, Scherf U, et al Exploration, normalization, and summaries of high density oligonucleotide array probe level data. Biostatistics 2003;4: 249–264. 10.1093/biostatistics/4.2.249 12925520

[22] Eide PW, Bruun J, Lothe RA, Sveen A. CMScaller: An R package for consensus molecular subtyping of colorectal cancer pre-clinical models. Sci Rep. 2017;7: 16618 10.1038/s41598-017-16747-x 29192179 PMC5709354

[23] Wang L, Wang. X. DEGseq: Identify differentially expressed genes from RNA-seq data. R package version 1.36.1. 10.1093/bioinformatics/btp612 19855105

[24] Szklarczyk D, Franceschini A, Wyder S, Forslund K, Heller D, Huerta-Cepas J, et al STRING v10: protein-protein interaction networks, integrated over the tree of life. Nucleic Acids Res. 2015 43(Database issue):D447–52. 10.1093/nar/gku1003 25352553 PMC4383874

[25] Ashburner et al Gene ontology: tool for the unification of biology. Nat Genet. 5 2000;25(1):25–9. 10.1038/75556 10802651 PMC3037419

[26] The Gene Ontology Consortium. The Gene Ontology Resource: 20 years and still GOing strong. Nucleic Acids Res. 1 2019;47(D1):D330–D338. 10.1093/nar/gky1055 30395331 PMC6323945

[27] GO Enrichment Analysis: Mi H, Huang X, Muruganujan A, Tang H, Mills C, Kang D, Thomas PD. PANTHER version 11: expanded annotation data from Gene Ontology and Reactome pathways, and data analysis tool enhancements. Nucleic Acids Res. 1 2017;45(D1):D183–D189. 10.1093/nar/gkw1138 27899595 PMC5210595

[28] DennisGlynnJr., ShermanBrad T., HosackDouglas A., YangJun, BaselerMichael W., LaneH. Clifford, et al DAVID: Database for Annotation, Visualization, and Integrated Discovery. Genome Biology 2003 4(5): P3 12734009

[29] Supek F, Bošnjak M, Škunca N, Šmuc T. REVIGO Summarizes and Visualizes Long Lists of Gene Ontology Terms. Plos One 2011;6: e21800 10.1371/journal.pone.0021800 21789182 PMC3138752

[30] Aguirre-Gamboa R, Gomez-Rueda H, Martínez-Ledesma E, Martínez-Torteya A, Chacolla-Huaringa R, Rodriguez-Barrientos A, et al SurvExpress: An online biomarker validation tool and database for cancer gene expression data using survival analysis. Plos One 2013;8, e74250 10.1371/journal.pone.0074250 24066126 PMC3774754

[31] Komor MA, Bosch LJ, Bounova G, Bolijn AS, Delis-van Diemen PM, Rausch C, et al Consensus molecular subtype classification of colorectal adenomas. J Pathol. 2018;246: 266–276. 10.1002/path.5129 29968252 PMC6221003

[32] Kotian S, Banerjee T, Lockhart A, Huang K, Catalyurek UV, Parvin JD. NUSAP1 influences the DNA damage response by controlling BRCA1 protein levels. Cancer Biol Ther. 2014;15: 533–543. 10.4161/cbt.28019 24521615 PMC4026076

[33] Liu Z, Guan C, Lu C, Liu Y, Ni R, Xiao M, et al High NUSAP1 expression predicts poor prognosis in colon cancer Author links open overlay panel. Pathology-Research and Practice 2018;214: 968–973. 10.1016/j.prp.2018.05.017 29853313

[34] Chen C, Zhao S, Karnad A, Freeman JW. The biology and role of CD44 in cancer progression: Therapeutic implications. J Hematol Oncol. 2018;11: 64 10.1186/s13045-018-0605-5 29747682 PMC5946470

[35] Assadian S, El-Assaad W, Wang XQ, Gannon PO, Barrès V, Latour M, et al p53 Inhibits angiogenesis by inducing the production of Arresten. Tumor and Stem Cell Biology 1 17, 2012 10.1158/0008-5472.CAN-11-2348 22253229

[36] Wang L, Yu J, Ni J, Xu XM, Wang J, Ning H, et al Extracellular matrix protein 1 (ECM1) is over-expressed in malignant epithelial tumors. Cancer Lett. 2003;200: 57–67. 10.1016/s0304-3835(03)00350-1 14550953

[37] Ma JC, Sun XW, Su H, Chen Q, Guo TK, Li Y, et al Fibroblast-derived CXCL12/SDF-1α promotes CXCL6 secretion and co-operatively enhances metastatic potential through the PI3K/Akt/mTOR pathway in colon cancer. World J Gastroenterol. 2017;23: 5167–5178. 10.3748/wjg.v23.i28.5167 28811711 PMC5537183

[38] Divella R, Daniele A, De Luca R, Simone M, Naglieri E, Savino E, et al Circulating levels of VEGF and CXCL1 are predictive of metastatic organotropismin in patients with colorectal cancer. Anticancer Res. 2017;37: 4867–4871. 10.21873/anticanres.11895 28870907

[39] Wang D, Jiao C, Zhu Y, Liang D, Zao M, Meng X, et al Activation of CXCL12/CXCR4 renders colorectal cancer cells less sensitive to radiotherapy via up-regulating the expression of surviving. Exp Biol Med. 2017;242: 429–435. 10.1177/1535370216675068 PMC5298539 27798120

[40] Fukumoto M, Kurisu S, Yamada T, Takenawa T. α-Actinin-4 enhances colorectal cancer cell invasion by suppressing focal adhesion maturation. PLoS One 2015;10: e0120616 10.1371/journal.pone.0120616 25860875 PMC4393021

[41] Shida D, Inoue S, Yoshida Y, Kodaka A, Tsuji T, Tsuiji M. Sphingosine kinase 1 is upregulated with lysophosphatidic acid receptor 2 in human colorectal cancer. World J Gastroenterol 2016;22: 2503–2511. 10.3748/wjg.v22.i8.2503 26937138 PMC4768196

[42] Cao Q, McIsaac SM, Stadnyk AW. Human colonic epithelial cells detect and respond to C5a via apically expressed C5aR through the ERK pathway. Am J Physiol Cell Physiol. 2012;302: C1731–C1740. 10.1152/ajpcell.00213.2011 22496247

[43] Wu Z, Huang X, Han X, Li Z, Zhu Q, Yan J, et al The chemokine CXCL9 expression is associated with better prognosis for colorectal carcinoma patients. Biomedicine & Pharmacotherapy 2016;78: 8–13. 26898419 10.1016/j.biopha.2015.12.021

[44] Stäubert C, Broom OJ, Nordström A. Hydroxycarboxylic acid receptors are essential for breast cancer cells to control their lipid/fatty acid metabolism. Oncotarget 2015;6: 19706–19720. 10.18632/oncotarget.3565 25839160 PMC4637315

[45] Elangovan S, Pathania R, Ramachandran S, Ananth S, Padia RN, Lan L, et al The niacin/butyrate receptor GPR109A suppresses mammary tumorigenesis by inhibiting cell survival. Cancer Res. 2014;74: 1166–1178. 10.1158/0008-5472.CAN-13-1451 24371223 PMC3944627

[46] Pal J, Patil V, Mondal B, Shukla S, Hegde AS, Arivazhagan A, et al Epigenetically silenced GNG4 inhibits SDF1α/CXCR4 signaling in mesenchymal glioblastoma. Genes Cancer. 2016;7: 136–147. 10.18632/genesandcancer.105 27382437 PMC4918951

[47] Thangaraju M, Cresci GA, Liu K, Ananth S, Gnanaprakasam JP, Browning DD, et al GPR109A is a G-protein-coupled receptor for the bacterial fermentation product butyrate and functions as a tumor suppressor in colon. Cancer Res. 2009;69: 2826–2832. 10.1158/0008-5472.CAN-08-4466 19276343 PMC3747834

[48] Zhai X, Xue Q, Liu Q, Guo Y, Chen Z. Colon cancer recurrence-associated genes revealed by WGCNA co-expression network analysis. Mol Med Rep. 2017;16: 6499–6505. 10.3892/mmr.2017.7412 28901407 PMC5865817

[49] Wang D, Wang H, Brown J, Daikoku T, Ning W, Shi Q, et al CXCL1 induced by prostaglandin E2 promotes angiogenesis in colorectal cancer. JEM 2006;203: 941. 10.1084/jem.20052124 PMC2118273 16567391

