# Supplementary figures and images for: Identification of modules and functional analysis in CRC subtypes by integrated bioinformatics analysis
Source: PLoS One. 2019 Aug 30;14(8):e0221772. doi: 10.1371/journal.pone.0221772 (PMC6716647; doi:10.1371/journal.pone.0221772)

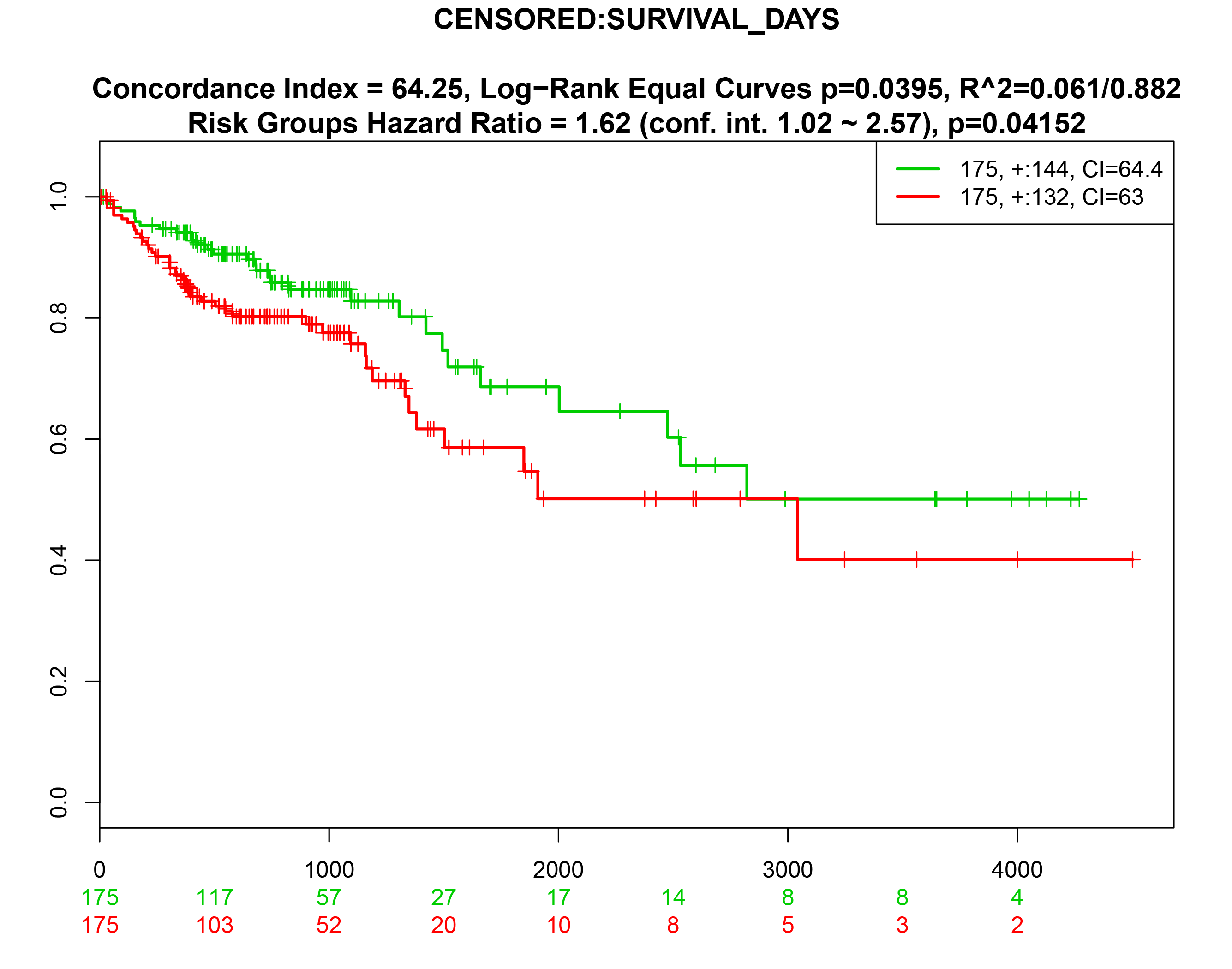

Supplement: S1 Fig — (TIF) [file pone.0221772.s002.tif]
